# Novel Methods for the Analysis of Serum NET Remnants: Evaluation in Patients with Severe COVID-19

**DOI:** 10.3390/ijms26052221

**Published:** 2025-02-28

**Authors:** Francesco Pisani, Caterina Porciani, Cristina Croia, Valentina Pucino, Agostino Virdis, Ilaria Puxeddu, Paola Migliorini, Federico Pratesi

**Affiliations:** 1 Clinical Immunology and Allergy Unit, Department of Clinical and Experimental Medicine, University of Pisa, 56126 Pisa, Italy; francesco.pisani@phd.unipi.it (F.P.); caterina.porciani@gmail.com (C.P.); cristina.croia@unipi.it (C.C.); valentina.pucino@unipi.it (V.P.); ilaria.puxeddu@unipi.it (I.P.); paola.migliorini@unipi.it (P.M.); 2 Geriatrics Unit, Department of Clinical and Experimental Medicine, University of Pisa, 56126 Pisa, Italy; agostino.virdis@unipi.it; 3 Department of Translational Medicine and NTMS, University of Pisa, 56126 Pisa, Italy

**Keywords:** neutrophil extracellular traps, NETs, myeloperoxidase, alpha-enolase, calprotectin, COVID-19 pneumonia

## Abstract

Neutrophil extracellular traps (NETs) are web-like structures composed of chromatin and proteins from neutrophil granules. Several studies highlight the heterogeneity of NETs, underscoring the challenges associated with their detection. In patients with COVID-19, high levels of NET fragments, called NET remnants, are detected in the circulation but also in alveoli and bronchioles. NET remnants are usually measured as complexes of DNA and myeloperoxidase (DNA−MPO). Taking advantage of proteomic data on NET composition, we developed new solid-phase assays to detect NET remnants, measuring complexes of DNA with alpha enolase (DNA−eno) or calprotectin (DNA−cal). The two assays were compared with the DNA−MPO test for the detection of in vitro-generated NET and serum NET remnants; all of them showed similar sensitivity in the detection of in vitro-generated NET. In an analysis of 40 patients with severe COVID-19 and 25 healthy subjects, the results of the three assays were highly correlated, and all detected significantly higher levels of NET remnants in patient sera. Moreover, the level of NET remnants correlated with impaired gas exchange and increased with the progressive decline of pulmonary function. The proposed assays thus represent a novel tool with which to evaluate NETosis; using antibodies to different NET constituents may allow their fingerprinting in different disorders.

## 1. Introduction

Neutrophil extracellular traps (NETs) were described for the first time in 2004 as extracellular web-like structures made of DNA, histones and several proteins from neutrophil cytoplasmatic granules that entrap microorganisms, containing the spread of infection [1]. NETs are formed during a process called NETosis, an active cell-death pathway different from other death pathways such as apoptosis and necrosis [2].

Acting on different neutrophil receptors, several stimuli trigger NET formation, leading to the activation of NADPH oxidase (NOX)-dependent or -independent pathways.

In this process, the key event is the production, by NOX or the respiratory chain of mitochondria, of reactive oxygen species that generate hydrogen peroxide [3].

Myeloperoxidase (MPO) converts hydrogen peroxide into hypochlorous acid and activates neutrophil elastase (NE), which degrades the nuclear membrane and cytoskeleton [4]. Proteolytic activity of MPO and NE, together with deimination of histones by activated peptidylarginine deiminase (PAD), allows chromatin decondensation [5]. Granular and cytoplasmic proteins associate with chromatin fibers, which are then released extracellularly.

In bacterial and fungal disorders, NETs are a critical mechanism of defense and also protect against many viral pathogens [6]. Viruses can induce NETosis directly or indirectly, and NETs contain infection, immobilizing and inactivating viral particles [7]. However, excessive NET formation can contribute to viral immunopathology, as clearly shown in COVID-19. In SARS CoV2-infected patients, the number of circulating neutrophils increases. In COVID-19 pneumonia, NETs are abundant in alveoli and bronchioles, but also in arterioles and in the interstitial compartment [8]. NETs have the ability to induce epithelial and endothelial damage, which is mediated predominantly by histones [9], impacting the initiation and progression of acute respiratory distress syndrome (ARDS) [10].

NET interactions with endothelial cells and platelets contribute to the formation of thrombi; this is another mechanism to contain the spread of infection that links innate immunity to coagulation and is known as immune thrombosis [11,12]. In COVID-19, it has been reported that NETs colocalize with platelets in thrombotic lesions of lung vessels and that increased NETosis is one of the factors that trigger widespread vascular injury. In fact, several studies demonstrate the role of NETosis in disease severity and the overall prognosis of COVID-19 patients [13].

The result of increased NET formation is the persistence in the peripheral blood of fragments derived from NETs, the NET remnants, which are made up of chromatin complexed with cytoplasmic proteins. The measurement of circulating NET remnants is of interest in inflammatory disorders, representing a simple tool with which to evaluate NETosis.

So far, the available methods to measure NET remnants are based on the detection of DNA complexed with myeloperoxidase or elastase. Most published data were obtained using a monoclonal anti-MPO as the capture antibody and a monoclonal anti-DNA as the detection antibody [14,15,16].

Taking advantage of what is known about NET composition, we propose new assays to detect NET remnants in the peripheral blood that are based on antibodies to two abundant NET components: alpha enolase and calprotectin.

## 2. Results

### 2.1. Detection of NET Generated In Vitro

**Figure 1:**
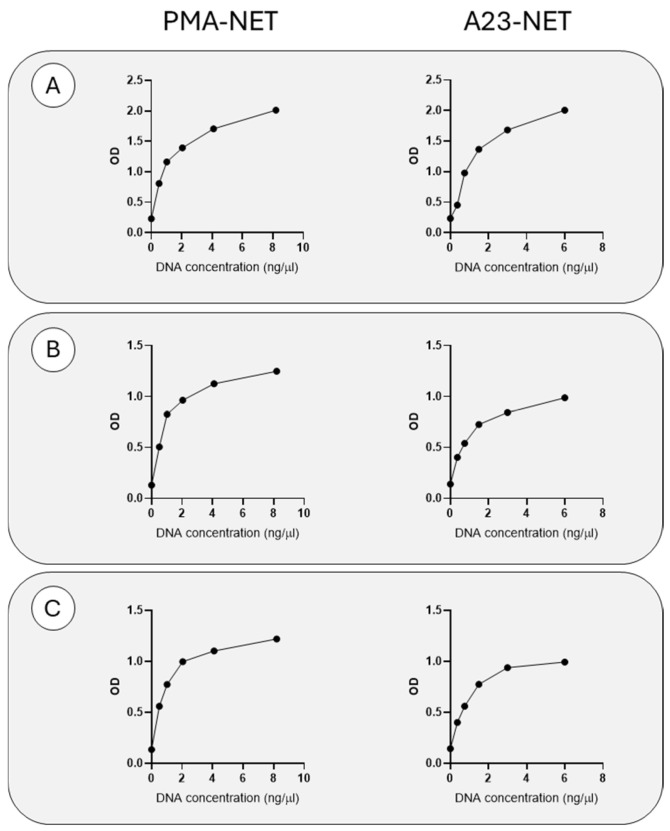
Detection of NETs formed in vitro. The figure reports the data obtained with increasing amounts of NETs from neutrophils stimulated by PMA or A23187, expressed as DNA concentration. The results are given as absorbance obtained in the MPO−DNA assay (**A**), enolase−DNA assay (**B**) and calprotectin−DNA assay (**C**).

Monoclonal anti-MPO, anti-enolase and anti-calprotectin antibodies were used to coat a polystyrene plate, as outlined in the Materials and Methods. In vitro PMA and A23187-generated NETs were used at two-fold dilution to generate a six-point standard curve. Figure 1 shows the results obtained in the three assays with the two NET preparations. All the assays detect NETs generated in vitro in a dose-dependent way.

### 2.2. Detection of NET Remnants in Sera

NET remnants were quantified by assays using anti-MPO, anti-enolase and anti-calprotectin antibodies in 25 healthy subjects and 40 COVID-19 sera.

**Figure 2:**
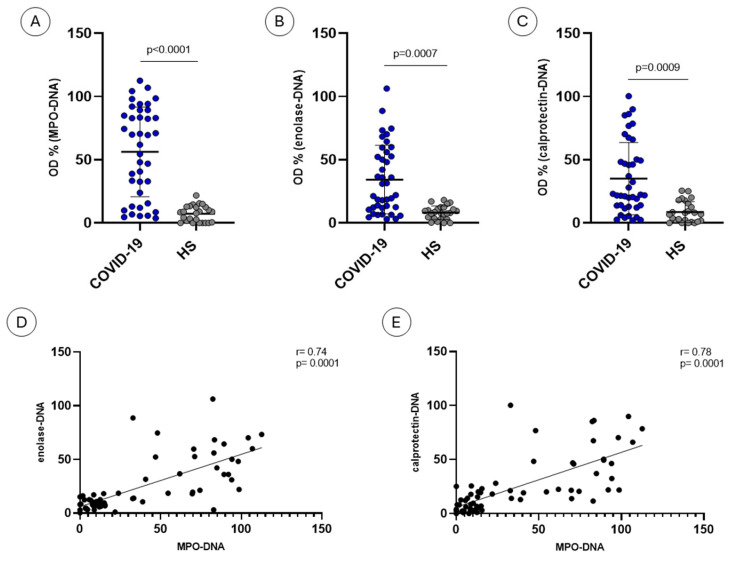
Levels of NET remnants and correlations among the three assays. Healthy subjects (HS) and COVID-19 sera were tested for MPO−DNA (**A**), enolase−DNA (**B**) and calprotectin−DNA (**C**) complexes. Correlations between MPO−DNA and enolase−DNA (**D**) and between MPO−DNA and calprotectin−DNA (**E**) were also observed. Data are expressed as percentages relative to an internal positive control.

The results, expressed as ratios to a positive control, are given in Figure 2. Significantly higher amounts of NET remnants were detected in COVID-19 sera as compared to controls by anti-MPO (Figure 2A, *p* < 0.0001), anti-alpha enolase (Figure 2B, *p* = 0.0007) and anti-calprotectin (Figure 2C, *p* = 0.0009).

In order to explore the relationship between levels of DNA–MPO complexes and levels of those involving alpha enolase or calprotectin, we analyzed the correlations among the levels of NET remnants detected by the three assays.

Levels of MPO−DNA complexes are correlated with the levels of enolase−DNA complexes (Figure 2D, *p* = 0.0001) and calprotectin−DNA complexes (Figure 2E, *p* = 0.0001).

### 2.3. NET Remnants and Clinical Features

**Figure 3:**
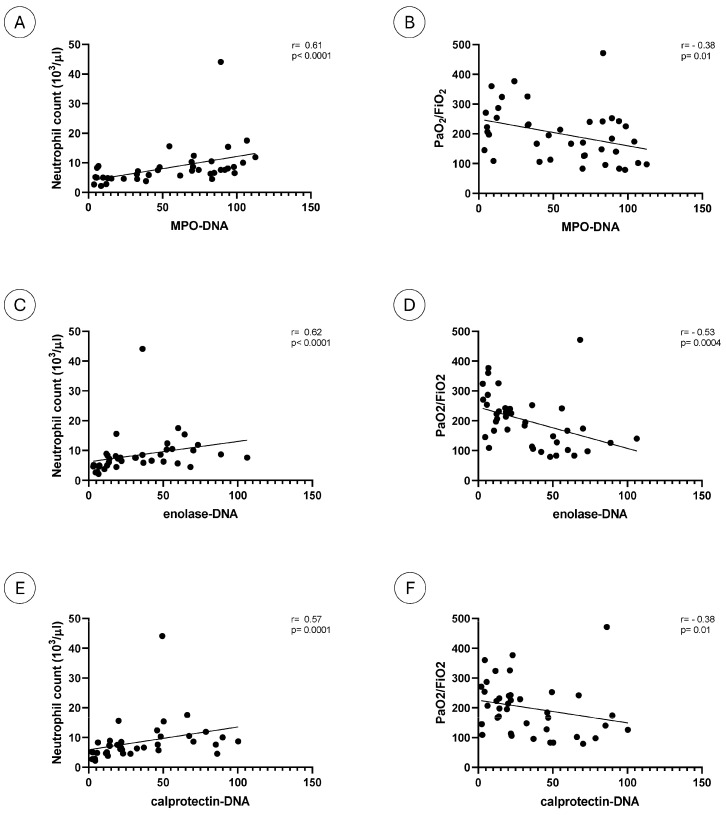
NET remnants and clinical parameters. The figure reports the correlations between neutrophil number and MPO−DNA (**A**), enolase−DNA (**C**) and calprotectin−DNA (**E**) complexes and between FiO_2_/PaO_2_ and MPO−DNA (**B**), enolase−DNA (**D**) and calprotectin−DNA (**F**) complexes.

Levels of NET remnants detected by the three assays were positively correlated with neutrophil number, with similar correlation coefficients (MPO−DNA *p* < 0.0001; Figure 3A; enolase−DNA *p* < 0.0001; Figure 3C; calprotectin−DNA *p* = 0.0001; Figure 3E). No correlations were observed with platelet number or CRP or LDH levels (Table 1).

We then analyzed the relationship between NET remnants and severity of lung involvement. A negative correlation was observed with the ratio of arterial oxygen partial pressure to fractional inspired oxygen, with identical correlation coefficients for MPO−DNA and calprotectin−DNA (*p* = 0.01; Figure 3B–F) and a higher correlation coefficient for enolase−DNA (*p* = 0.0004; Figure 3D).

Patients were then classified according to the presence and severity of ARDS in four classes (no ARDS and mild, moderate and severe ARDS) based on the FiO_2_/PaO_2_ ratio according to the Berlin definition [17].

**Figure 4:**
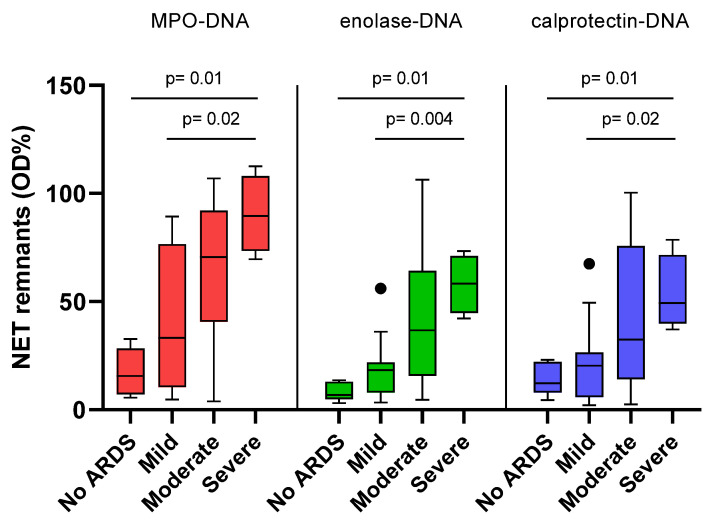
NET remnants and lung involvement, Distribution of levels of NET remnants in four classes of severity of lung involvement (no ARDS n° = 5; Mild n° = 10; Moderate n° = 19; Severe n° = 4). Levels of NET remnants increase according to ARDS severity, with significant differences between severe ARDS and mild ARDS and between severe ARDS and absence of ARDS. Black dots indicate outliers.

The levels of NET remnants showed a progressive increase with worsening of lung function: they increase according to ARDS severity, with significant differences between severe ARDS and mild ARDS and between severe ARDS and absence of ARDS (Figure 4).

## 3. Discussion

The detection of NET remnants in sera by solid-phase assays has been proposed as a quantitative, objective and specific method to evaluate NETosis in vivo [18]. In this context, the measurement of DNA−MPO or DNA−elastase complexes is the most frequently used approach. In this paper, we propose two novel assays based on the measurement of complexes of DNA with alpha enolase or calprotectin.

Proteomic analysis of NET indicates that both alpha enolase and calprotectin are abundant proteins in NETs produced in response to different stimuli [19,20,21]. Stemming from this observation, we developed two immunoassays that employ monoclonal antibodies specific for alpha enolase or calprotectin.

The monoclonal anti-alpha enolase antibody IgG 2a 276/3 was obtained from mice immunized with recombinant human alpha enolase. The Mab 276/3 can immunoprecipitate the cytoplasmic or membrane form of the enzyme [22], and the recognized epitope lies in sequence 77–96 of the enzyme.

NET generated in vitro, as well as serum NET remnants, was detected by all three immunoassays. Higher levels were found with the MPO−DNA assay, but the results obtained with the different assays show a high correlation coefficient.

PMA and A23187 are reported as highly efficient inducers of NETosis. However, it is known that NETs produced in response to different stimuli contain a common core of proteins but also contain proteins that are differentially expressed [18]. PMA−NETs are the most heterogeneous in term of protein composition and are very similar to A23187-induced NETs.

The standardization of the methods for the detection of NET remnants proved to be difficult because of the cross-reactivities of the antibodies and the presence of heterophile antibodies [23]. Further complexity is created by the extreme heterogeneity of NET remnants in terms of size and conformation. It has been reported that also the storage of samples may modify NET structure and consequently modify reactivity in a given assay [24].

Thus, NET constituents may be exposed to different extents and may be differentially recognized by the reagents used in the assays. Moreover, NET composition may differ in different disorders, especially in terms of the type and amount of post-translationally modified proteins: for example, oxidized enolase is highly enriched in NETs from SLE patients with active nephritis [25].

The results we obtained are consistent with NET heterogeneity and complexity. Proteomic analysis of NETs has identified MPO as a very abundant constituent that is contained in NETs in greater amounts than calprotectin or alpha enolase [19,20]. Accordingly, the anti-MPO antibody detects NET remnants in greater amounts than do the anti-calprotectin or anti-enolase antibodies.

In severe COVID-19, the three assays detect high levels of NET remnants in sera, as previously described [26]. In this disease, elevated levels of blood neutrophils predict severe manifestations of the disease [27]. However, medical therapies, specifically steroids, affect neutrophil number, and most of the patients included in the study were treated with multiple drugs, including steroids. Despite this confounding factor, the strong correlation between neutrophil number and levels of NET remnants is of interest; according to several studies, these values are elevated in severe forms of COVID-19 [28,29]. We also found high levels of NET remnants in patients with severe COVID-19 pneumonia, as shown by the strong correlation with impaired gas exchange. Increased levels in patients with progressive decline of pulmonary function further support the role of NET remnants as biomarkers of severe lung involvement. Under this respect, NET remnants detected by anti-enolase or anti calprotectin show a higher ability to discriminate mild ARDS vs severe ARDS, as compared with anti MPO. This result, if confirmed in other cohorts of patients, suggests the utility of measuring NET remnants by different assays when evaluating lung involvement in COVID-19.

On the whole, the proposed assays represent a novel tool with which to evaluate NETosis. The high correlation coefficients among the assays suggest that they all detect NET fragments, although to a different extent. This differential recognition may reflect pathogenic potential (see the high correlation coefficient between enolase−DNA complexes and lung damage). This observation should be further tested in other disorders characterized by increased NETosis and/or reduced NET clearance. Thus, the use of antibodies against different NET constituents may allow a better characterization of NET remnants in different disorders.

## 4. Materials and Methods

### 4.1. Study Population

Serum samples were obtained from 40 patients affected by a severe form of COVID-19 (19 females and 21 males; mean age 64, range: 42–83) and 25 healthy subjects (13 females and 12 males; mean age 44, range 27–61) and stored immediately at −80 °C until analysis.

The 40 consecutive highly symptomatic COVID-19 patients were recruited at the Intensive Care Unit (ICU) of the AOU Pisana, and data were collected from clinical charts (Table 2). In particular, neutrophil count, C-reactive protein (CRP), interleukin-6 (IL-6), D-dimer, lactate dehydrogenase (LDH), procalcitonin (PCT), platelet count (PLT), partial oxygen pressure (PaO_2_), partial carbon dioxide pressure (PaCO_2_), fraction of inspired oxygen (FiO_2_) and FiO_2_/PaO_2_ ratio were taken into account. Comorbidities (obesity, hypertension, diabetes and dyslipidemia) were also evaluated.

Informed consent was obtained from all the subjects, and the study was approved by the Ethical Committee of the Azienda Ospedaliera Universitaria Pisana (protocol N° 17522).

### 4.2. Neutrophil Isolation and In Vitro Generation of NETs 

Neutrophils were isolated from fresh ethylenediaminetetraacetic acid (EDTA)-anticoagulated peripheral blood from four different healthy donors and stimulated to induce NET release with two different stimuli, as previously described [15], but with minor modifications. Briefly, fresh blood was diluted in Dulbecco’s phosphate-buffered saline (D-PBS) (Sigma-Aldrich, St. Louis, MO, USA), carefully stratified over a double gradient and centrifuged at 700× *g* for 30 min at room temperature with no acceleration or brake.

Neutrophil were seeded in Hank’s balanced salt solution (HBSS) with CaCl_2_ 2 mM (Sigma Aldrich, St. Louis, MO, USA) at a concentration of 10 × 10^6^ cells/plate and treated with 100 nM phorbol 12-myristate 13-acetate (PMA) (Sigma Aldrich, St. Louis, MO, USA) or 2 µM calcium ionophore A23187 diluted in HBSS CaCl_2_ 2 mM for 3 h at 30 °C under 5% CO_2_.

After the stimulation, the dishes were gently washed twice with D-PBS and incubated with 100 U/mL DNase I (Sigma Aldrich, St. Louis, MO, USA) in D-PBS CaCl_2_ 2 mM for 30 min at 37 °C. Next, the DNase activity was blocked by the addition of EDTA 5 mM (final concentration). The samples were centrifuged for 10 min at 3000 rpm, and supernatants containing PMA- or A23187-derived NETs were separately pooled and frozen in aliquots at −80 °C.

NETs were quantified based on their DNA content using the Qubit^TM^ dsDNA BR assay kit (Thermo Fisher Scientific, Waltham, MA, USA) according to the manufacturer’s recommendations. DNA concentration was 8.2 ng/mL for PMA-NET and 6 ng/mL for A23-NET.

### 4.3. Detection of Serum NET Remnants

NET remnants were detected in patient sera and healthy controls with an in-house sandwich enzyme-linked immunosorbent assay (ELISA).

To quantify MPO−DNA complexes, 96-well polystyrene Nunc Maxisorp plates (Sigma Aldrich, St. Louis, MO, USA) were coated overnight at 4 °C with 5 µg/mL of mouse anti-human MPO monoclonal antibody (Biorad, Hercules, CA, USA) diluted in 0.1 M carbonate buffer, pH 9.6.

For the detection of alpha enolase−DNA complexes, plates were coated overnight with 1 µg/mL of mouse anti-human alpha enolase monoclonal antibody (276/3) diluted in 0.1 M carbonate buffer, pH 9.6.

For the detection of calprotectin–DNA complexes, plates were coated overnight at 4 °C with 5 µg/mL of mouse anti-human calprotectin monoclonal antibody (Eurospital, Trieste, Italy) diluted in 0.1 M carbonate buffer, pH 9.6.

The plates were washed once with PBS 0.05% Tween-20 (wash buffer) and blocked with PBS 1% BSA (Sigma Aldrich, St. Louis, MO, USA) (MPO-coated plates) or PBS 1% porcine gelatine (enolase and calprotectin) for 1 h under agitation at room temperature.

Controls (PMA and A23187 in vitro-generated NETs) were diluted 1:2, and serum samples were diluted 1:10 in PBS 1% BSA 0.05% Tween-20 (or PBS−gelatin 0.5% Tween 0.05% for calprotectin and enolase). After 2 h of incubation at room temperature under agitation, three washes were performed as indicated above.

A monoclonal HRP-labeled anti-DNA antibody was diluted in PBS 1% BSA 0.05% Tween-20 (or PBS−gelatin 0.5% Tween 0.05% for calprotectin and enolase) and added to the plate. After 2 h of incubations and washings as above, 3,3′,5,5′-tetramethylbenzidine (TMB) substrate (Sigma Aldrich, St. Louis, MO, USA) was added. Next, a 2 M sulfuric acid stop solution (Sigma Aldrich, St. Louis, MO, USA) was added. Absorbance was measured at 450 nm with an iMark^TM^ (Biorad, Hercules, CA, USA) microplate reader.

Results are expressed as the percentage obtained by dividing the optical densities obtained in the samples by those of the in vitro-generated NETs at 4 ng/mL. All values were blank-corrected.

### 4.4. Statistical Analysis

Statistical analyses were performed using GraphPad Prism software (Version 8.0.1). Data are shown as medians with standard deviation; A Mann−Whitney U test was used to assess the differences between two groups, and an ANOVA Kruskal−Wallis test was used for multiple comparisons. Correlations were calculated using a non-parametric Spearman analysis. Two-sided *p*-values ˂ 0.05 were considered statistically significant.

## Figures and Tables

**Table 1:** Correlations between clinical parameters and NET remnants. (n.s: not significant).

	PaO_2_/FiO_2_	Neutrophils Count (n° × 10^3^/µL)	CRP (mg/dL)	D-Dimer (ng/mL FEU)	IL-6 (pg/mL)	PLT (n° × 10^3^/µL)	LDH (U/L)	PCT (ng/mL)
**MPO-DNA** **(%)**	*p* = 0.02	*p* = 0.006	n.s	n.s	n.s	n.s	n.s	n.s
**enolase-DNA** **(%)**	*p* = 0.0004	*p* < 0.0001	n.s	n.s	n.s	n.s	n.s	n.s
**calprotectin-DNA** **(%)**	*p* = 0.01	*p* = 0.0001	n.s	n.s	n.s	n.s	n.s	n.s
**PaO** **2** **/FiO** **2**	-	*p* = 0.004	*p* = 0.052	n.s	n.s	n.s	*p* = 0.02	n.s
**Neutrophils count** **(n° × 10^3^/µL)**	*p* = 0.004	-	n.s	n.s	n.s	n.s	n.s	n.s
**CRP** **(mg/dL)**	*p* = 0.052	n.s	-	n.s	*p* = 0.02	n.s	*p* = 0.006	*p* < 0.0001
**D-dimer** **(ng/mL FEU)**	n.s	n.s	n.s	-	n.s	n.s	*p* = 0.005	*p* = 0.008
**IL-6** **(pg/mL)**	n.s	n.s	*p* = 0.02	n.s	-	n.s	n.s	n.s
**PLT** **(n° × 10^3^/µL)**	n.s	n.s	n.s	n.s	n.s	-	n.s	n.s
**LDH** **(U/L)**	*p* = 0.02	n.s	*p* = 0.006	*p* = 0.05	n.s	n.s	-	*p* = 0.04
**PCT** **(ng/mL)**	n.s	n.s	*p* < 0.0001	*p* = 0.008	n.s	n.s	*p* = 0.04	-

**Table 2:** Demographic and clinical characteristics of healthy subjects and patients with COVID-19.

Demographic characteristics	
Healty subjects (n°)	25
Mean age—yr (range)	44 (27−61)
Sex (M/F)	12/13
Patients (n°)	40
Mean age—yr (range)	64 (42−83)
Sex (M/F)	21/19
**Comorbidities**	
Obesity n° (%)	8/40 (20%)
Hypertension n° (%)	14/40 (47.5%)
Diabetes n° (%)	9/40 (22.5%)
Dyslipidemia n° (%)	4/40 (10%)
**Clinical parameters**	
PaO_2_/FiO_2_	199.6 ± 90.3
Neutrophil count (n° × 10^3^/µL) (mean ± SD)	8.5 ± 6.8
CRP (mg/dL) (mean ± SD)	10.3 ± 8.3
IL-6 (pg/mL) (mean ± SD)	26.1 ± 28.3
D-dimer (ng/mL FEU) (mean ± SD)	1871 ± 4435
LDH (U/L) (mean ± SD)	395.2 ± 181.8
PCT (ng/mL) (mean ± SD)	0.8 ± 2.2
PLT (n° × 10^3^/µL) (mean ± SD)	231.8 ± 113

## Data Availability

All the data are contained within the article.
